# Therapeutically targeting proinflammatory type I interferons in systemic lupus erythematosus: efficacy and insufficiency with a specific focus on lupus nephritis

**DOI:** 10.3389/fimmu.2024.1489205

**Published:** 2024-10-16

**Authors:** Benjamin Lai, Shue-Fen Luo, Jenn-Haung Lai

**Affiliations:** ^1^ Department of Neurology, Chang Gung Memorial Hospital, Taoyuan, Taiwan; ^2^ Division of Allergy, Immunology, and Rheumatology, Department of Internal Medicine, Chang Gung Memorial Hospital, Tao-Yuan, Taiwan; ^3^ Graduate Institute of Medical Science, National Defense Medical Center, Taipei, Taiwan

**Keywords:** systemic lupus - erythematosus, type I interferon (IFN), lupus nephritis, immunopathogenesis, treatment

## Abstract

Type I interferons (IFN-Is) are important players in the immunopathogenesis of systemic lupus erythematosus (SLE). Pathogenic events in patients with SLE are potent triggers of IFN-I induction, yet IFN-I may induce or initiate the immunopathogenesis leading to these events. Because blocking IFN-I is effective in some clinical manifestations of SLE patients, concerns about the efficacy of anti-IFN-I therapy in patients with lupus nephritis remain. Tissues from kidney biopsies of patients with lupus nephritis revealed infiltration of various immune cells and activation of inflammatory signals; however, their correlation with renal damage is not clear, which raises serious concerns about how critical the role of IFN-I is among the potential contributors to the pathogenesis of lupus nephritis. This review addresses several issues related to the roles of IFN-I in SLE, especially in lupus nephritis, including (1) the contribution of IFN-I to the development and immunopathogenesis of SLE; (2) evidence supporting the association of IFN-I with lupus nephritis; (3) therapies targeting IFN-I and IFN-I downstream signaling molecules in SLE and lupus nephritis; (4) findings challenging the therapeutic benefits of anti-IFN-I in lupus nephritis; and (5) a perspective associated with anti-IFN-I biologics for lupus nephritis treatment. In addition to providing clear pictures of the roles of IFN-I in SLE, especially in lupus nephritis, this review addresses the lately published observations and clinical trials on this topic.

## 1 Introduction

### 1.1 The association between type I interferon and systemic lupus erythematosus

Systemic lupus erythematosus (SLE) is an autoimmune disease, and its worldwide prevalence and annual incidence are 2.9 to 241 patients per 100,000 people and 0.3 to 23.2 patients per 100,000 person-years, respectively (1). Patients with SLE present with variable disease severity and tend to have multiple organ involvement (2). For example, patients with SLE may present only with fatigue and mild skin lesions or life-threatening manifestations, such as neuropsychiatric, hematologic, cardiovascular or renal disorders. The etiology of SLE remains unclear and is generally considered multifactorial; both genetic and environmental factors are major contributing factors to SLE for most patients (3). The hallmark of the disease is the production of a variety of autoantibodies against various antigens derived from the cell membrane (such as antiphospholipid and anticardiolipin autoantibodies), cytosol (such as anti-SSA and anti-SSB autoantibodies) or nucleus (anti-double-stranded DNA autoantibodies). In addition, patients with SLE carry autoantibodies against mitochondrial components and infectious pathogens, such as virus components (4–6). By binding autoantibodies to these different antigens, immune complexes form and deposit in different tissues and organs, leading to organ damage and malfunction and various clinical manifestations. Earlier studies demonstrated that the immune complex containing nucleic acids released from necrotic or apoptotic cells is a potent inducer of Type I IFN (IFN-I comprising both IFN-α and IFN-β) secretion from plasmacytoid dendritic cells (pDCs) (7). Nucleic acids from released neutrophil extracellular traps (NETs) are also important inducers of IFN-I (8). Several mechanisms, such as the activation of toll-like receptors (TLRs), pattern recognition receptors (PRRs) and the mitochondrial machinery in response to pathogen-associated molecular patterns (PAMPs) from bacterial or virus infection or endogenously generated damage-associated molecular patterns (DAMPs), are involved in the induction of IFN-I (9–11).

The IFN-I family consists of the IFN-α, IFN-β, IFN-δ, IFN-ϵ, IFN-κ, and IFN-ω subgroups, and 13 subtypes of IFN-α have been identified. Through binding to IFN-α receptor 1 (IFNAR1) and 2 (IFNAR2), which are universally expressed in all nucleated cells, IFN-I mediates its effects by stimulating Janus kinase (JAK)/signal transducer and activator of transcription proteins (STAT) family proteins, whose activation triggers autoimmunity in SLE (12). After the binding of IFN-I to IFN receptors, conformational changes in IFN receptors activate JAK1 and tyrosine kinase 2 (TYK2), which are constitutively associated with IFN receptors. Four JAK family members comprise JAK1–3 and TYK2 nonreceptor tyrosine kinases (13). Upon activation, JAK1 and TYK2 then transphosphorylate themselves and phosphorylate IFN receptors, which creates docking sites for the recruitment of STAT1 and STAT2. The phosphorylation of STATs by JAKs causes dimerization and subsequent interaction with the transcription factor interferon regulatory factor 9 (IRF9) to form an interferon stimulated gene factor 3 (ISGF3) complex that translocates from the cytosol to the nucleus and mediates the transcriptional activation of IFN-stimulated genes (ISGs) by binding to IFN-stimulated response elements (ISREs). A significant correlation was observed between ISG expression and several immunological events, such as immune cell activation, cell secretion, and pathogen infection (14). In patients with SLE, aberrant activation of IFN-I signaling, such as increased expression of STAT1, constitutive phosphorylation of JAK1 and STAT2, and an exaggerated response to IFN-β stimulation in immune cells, can be readily observed (10).

Because the generation of various autoantibodies, such as anti-double stranded DNA antibodies (anti-dsDNA Abs), is a critical process in the immunopathogenesis of SLE, IFN-I and IFN-I signaling facilitate the differentiation of extrafollicular B cells into short-lived antibody-forming cells (15, 16). IFN-I can promote the maturation of myeloid DCs, which promote the proliferation and differentiation of autoreactive T and B lymphocytes as well as the production of various cytokines, such as interleukin (IL)-6, IL-15, B lymphocyte stimulators, chemokines, and autoantibodies, and the formation of immune complexes that further trigger and exacerbate IFN-I autoimmunity through autocrine effects (17–19). IFN-I may also affect chromatin remodeling and result in increased global epigenetic modifications, such as DNA hypomethylation and histone acetylation (20). Interferonopathy can be reflected in earlier clinical observations revealing that a population of patients with cancer or virus infection develop various autoantibodies after treatment with IFN-α (21). Several typical characteristics of SLE emerged in a 53-year-old female with cryoglobulinemic vasculitis associated with hepatitis C virus infection after receiving IFN-α treatment (22). Indeed, several autoimmune disorders may arise from IFN-α treatment, with a frequency estimated to range from 4% to 19% (23). Although IFN-I is very low or undetectable in the serum of most SLE patients, a significantly elevated IFN-I signature is detected in various organ systems, such as immune cells, the synovium, the kidney, and the skin, in approximately 60% of patients with SLE (17, 24). Studies also suggest that both the serum IFN-α level and the ISG score are equally effective at evaluating disease activity in patients with SLE (25).

Plasmacytoid DCs that express the IFN-I-inducible protein MxA accumulate in the skin of nearly all patients with cutaneous lupus erythematosus skin lesions (14/15), which suggests that these cells locally produce IFN-I (26). A strong correlation between IFN-I signaling and cutaneous manifestations and anti-dsDNA Ab formation is also observed in childhood-onset SLE (27). Single-cell RNA sequencing analysis was used to analyze the characteristics of ISGs in peripheral blood mononuclear cells (PBMCs) from SLE patients, and the results were compared with those from healthy individuals. Deng et al. reported that high ISGs were mainly expressed in CD14+ monocytes, CD1c− DCs, neutrophils, and low-density granulocytes (LDGs) (14). In addition to monocytes and DCs, other non-hematopoietic cells, such as keratinocytes, renal tubular cells, glial cells, synovial stromal cells and other tissue cells, can produce IFN-I under different inflammatory conditions (28, 29). Although the extrarenal source of IFN-I is critical in the immunopathogenesis of lupus nephritis (30), importantly, local resident cells, such as mesangial cells, podocytes, tubular epithelial cells and endothelial cells, rather than infiltrating immune cells and hematologic cells, are the major sources of IFN-α in the kidney and are responsible for IFN-α-mediated renal damage in SLE patients (28, 31, 32).

Although the association between IFN-I and SLE has been widely discussed in many reports (33–37), in addition to including some recently published studies in this aspect, we focused more on the potential challenges associated with IFN-I blockade therapy in the treatment of lupus nephritis.

## 2 IFN-I as an initiator that triggers immune responses in SLE

Although the IFN-I signature can be widely detected in various organs in patients with SLE and an association between these two factors exists (38), whether IFN-I directly contributes to disease development or the pathogenesis of the involved organ systems in SLE patients remains unclear (39, 40). Among the 47 genetic variants associated with SLE, more than half (27/47, 57%) are associated with IFN-I production or signaling (41). In individuals at risk of autoimmune connective tissue diseases (defined as antinuclear antibody (ANA) positive; ≤1 clinical SLE criterion; symptom duration <12 months and treatment-naïve individuals), studies suggest that two ISG expression scores in blood and skin samples effectively predict progression from ANA positivity to autoimmune connective tissue diseases (42). Because genetic factors contribute to the development of SLE, several gene variants, which are mostly located in noncoding regions and can regulate cellular functions related to transcriptional activity, splicing, mRNA stability and epigenetic modifications, are highly associated with the induction of IFN-I signals and carry a greater risk of SLE development (43). Among the 13 IFN-α subtypes, subtypes 1, 2 and 5 are strongly associated with SLE (44). Given that IFN-I is an potent inducer of STAT4 in immune responses including SLE (45, 46), studies examining the association between STAT4 variants and SLE revealed that naïve CD4+ T cells from healthy donors bearing nonrisk alleles but not risk alleles downregulated STAT4 in response to IL-12. Cells from healthy donors with risk alleles presented increased active STAT4 and increased IFN-γ production (47). Carriers of the risk variant exhibited exaggerated CD4+ proinflammatory capacities that, in the context of SLE, contributed to more severe disease (47). High levels of STAT4 expression in T cells caused by genetic manipulation *in vivo* also enhance glomerulonephritis in mice (47).

IFN-I is an important factor that triggers very early events of tissue inflammation, which can be highlighted in the immunopathogenesis of skin lesions in patients with SLE. Exposure to ultraviolet B (UVB) irradiation increased IFN-α mRNA expression in mouse skin, likely from skin-infiltrating pDCs, and this effect was more pronounced in lupus-prone MRL/lpr mice (48). Because photosensitivity is one of the characteristics of SLE patients, IFN-α mediates UV light-induced keratinocyte apoptosis in a caspase-8-dependent manner (49). Braunstein et al. reported a significant correlation between the expression levels of five IFN-I-regulated genes and the severity of skin manifestations in SLE patients with subacute cutaneous lupus erythematosus (SCLE) and discoid lupus erythematosus (DLE) (50); these genes included lymphocyte antigen 6 complex (LY6E), 2’,5’-oligoadenylate synthetase 1, OAS1, 2’,5’-oligoadenylate synthetase-like (OASL), ISG15 and myxovirus resistance 1 (MXI1). In addition to affecting the skin, IFN-I may influence the immunopathogenesis of many different organ systems, such as the musculoskeletal system, vascular system, hematopoietic system, renal system, and central nervous system (40). Together with many more undiscussed examples illustrating the association of IFN-I with SLE characteristics, these observations suggest the crucial roles of IFN-I in initiating immune responses and triggering disease development (29). Noticeably, the immunopathogenesis of skin lesions in patients with SLE serves as one of the examples explaining how a combination of genetic and environmental factors may contribute to the development of SLE, where IFN-I play critical roles (51). In considering that lupus nephritis remains one of the major challenging and difficult-to-treat organ disorders in patients with SLE, we used more volume to discuss the involvement of IFN-I in the immunopathogenesis of lupus nephritis.

## 3 IFN-I and lupus nephritis

### 3.1 Cellular effects of IFN-I in the kidney

**Figure 1:**
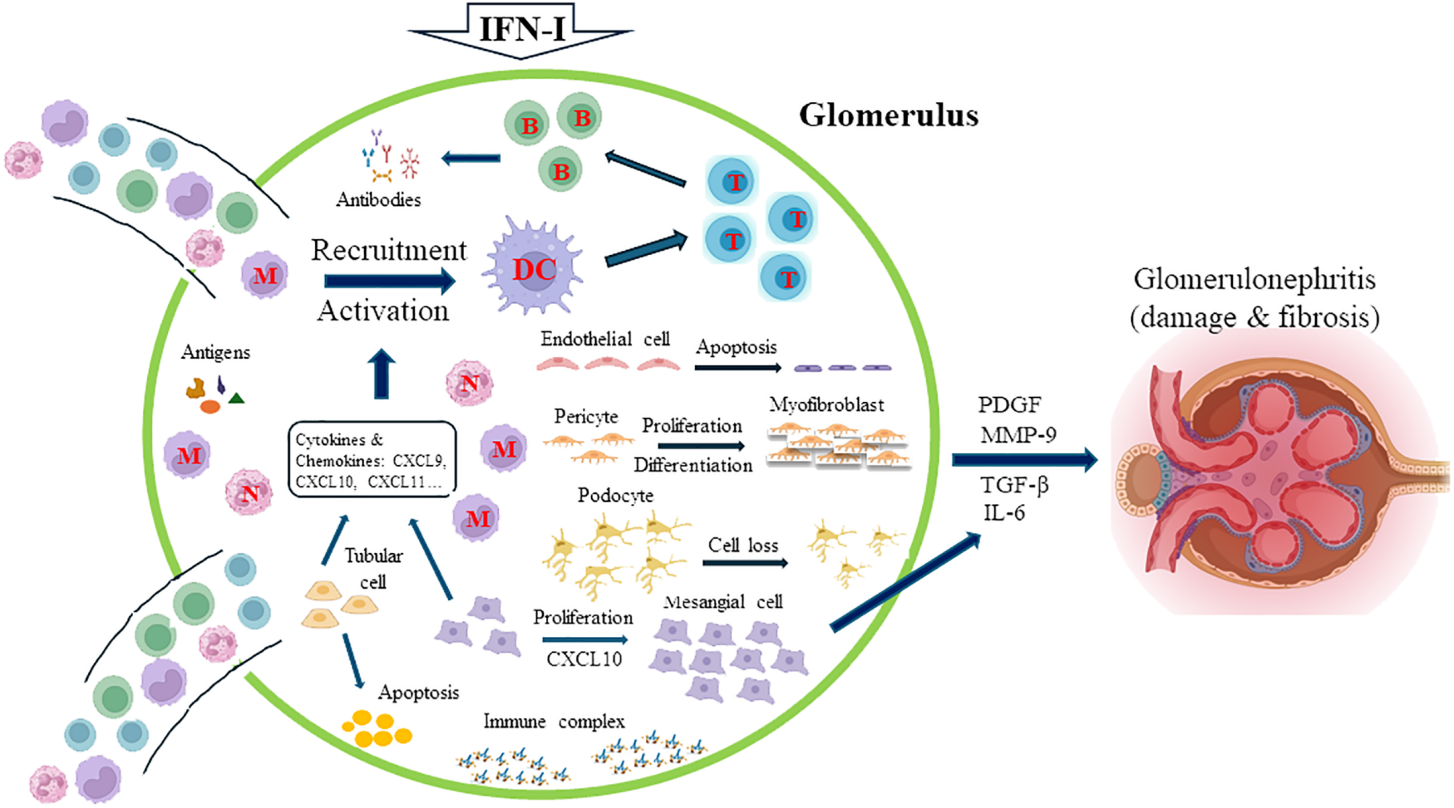
Major IFN-I-mediated effects on infiltrating and resident kidney cells. IFN-I induces the release of cytokines and chemokines, such as CXC chemokine ligand 9 (CXCL9), CXCL10, and CXCL11, from resident cells in the kidney, which recruit leukocytes into the kidney. Some of these mediators, such as CXCL10, cause the proliferation of mesangial cells, which, aggravated by the effects of IFN-I, produce matrix metalloproteinase-9 (MMP-9) and transforming growth factor (TGF)-β1, leading to the formation of fibrosis in the glomerulus. The activation and maturation of dendritic cells (DCs) promote the subsequent activation of T and B cells. IFN-I directly or indirectly regulates the formation of neoantigens, autoantibodies, and immune complexes. Critically, IFN-I increases podocyte loss, prevents podocyte repair and proper replacement, and suppresses the differentiation of podocyte progenitors into mature podocytes. By inducing the apoptosis of tubular epithelial cells and promoting capillary pericyte proliferation and differentiation into myofibroblasts, IFN-I, through platelet-derived growth factor (PDGF) and TGF-β1, also promotes kidney fibrosis. The proapoptotic effects of IFN-α in endothelial progenitor cells and myelomonocytic circulating angiogenic cells result in an imbalance between endothelial cell damage and repair. M, monocyte; N, neutrophil; T, T cell; B, B cell.

Many different triggers, such as nucleic acids or immune complexes, can induce IFN-I secretion from intrinsic renal cells and intrarenal infiltrating immune cells, and more than 200 ISGs are induced in a coordinated manner as a result of an autocrine-activation loop (52). Although the net influence of IFN-I on human glomerular diseases remains unclear, IFN-I may exert its effects on the kidney through two mechanisms: IFN-I can directly affect the biology and function of resident cells such as podocytes, mesangial, endothelial, and parietal epithelial cells in the kidney, and IFN-I may also indirectly activate immune cells or resident cells to produce proinflammatory mediators to cause renal damage (53). Several IFN-I-mediated effects on infiltrating and resident cells are summarized in **Figure 1**. IFN-I is a potent inducer of the production of cytokines and chemokines, such as CXC chemokine ligand 9 (CXCL9), CXCL10, and CXCL11, by resident cells in the kidney, and these medicators serve as strong chemoattractants to recruit leukocytes into the kidney (53). By inducing the expression of MHC class II and costimulatory molecules and driving dendritic cell maturation, IFN-I can indirectly prime and activate T cells (54–56). IFN-I plays direct or indirect roles in processes such as the formation of neoantigens, autoantibodies, and immune complexes (52, 53). IFN-I can induce the production of CXCL10, which causes the proliferation of mesangial cells. These cells produce matrix metalloproteinase-9 (MMP-9) and transforming growth factor (TGF)-β1, and this production is exacerbated by IFN-I to lead to the formation of fibrosis in the glomerulus (57). Importantly, IFN-I exerts several damaging effects on podocytes, including causing podocyte loss, preventing podocyte repair and proper replacement, and suppressing the differentiation of podocyte progenitors into mature podocytes (58). By inducing the apoptosis of tubular epithelial cells and promoting capillary pericyte proliferation and differentiation into myofibroblasts, IFN-I, through platelet-derived growth factor (PDGF) and TGF-β1, promotes kidney fibrosis (59). Furthermore, studies by Denny et al. demonstrated the proapoptotic effects of IFN-α in endothelial progenitor cells and myelomonocytic circulating angiogenic cells, which resulted in an imbalance between endothelial cell damage and repair (60).

The treatment of lupus nephritis remains challenging. Although medications such as glucocorticoids, cyclophosphamide, cyclosporin, mycophenolate mofetil, and azathioprine are helpful for many SLE patients, up to 40% of SLE patients remain unresponsive to the treatment and progress to chronic kidney disease over the course of the disease (61–63), and about 10% of those patients may finally reach to the status of end-stage renal disease (64, 65). The successful use of biological agents and small molecule inhibitors in patients with rheumatoid arthritis has inspired clinicians and scientists to develop more effective medications than conventional synthetic disease-modifying antirheumatic drugs (csDMARDs) for patients with SLE. Thus, there is an unmet need to identify therapeutically useful biological or small-molecule targets for more effective treatment of lupus nephritis. Accordingly, IFN-I serves as a potential target for therapeutics for lupus nephritis.

### 3.2 Effects of IFN-I in lupus animal models

Several mouse models of SLE that present with various clinical features of SLE have been developed, such as isoprenoid alkane 2, 6, 10, and 14 tetramethylpentadecane (pristane)-induced lupus mice; BXSB mice; F1 hybrids between New Zealand black and New Zealand white mice (NZB/W F1); and MRL/lpr mice. Among these animal models, MRL/lpr mice have gained the most popularity because of the spontaneous development of wider presentations of SLE characteristics, including antinuclear Ab (ANA)/anti-ds DNA Ab, low complement levels, lupus nephritis, neurological manifestations, skin lesions and joint inflammation (66). To directly evaluate the effects of IFN-I in lupus nephritis, animals can be induced with recombinant IFN-α, infected with an adenovirus expressing IFN-α or treated with an augmenting IFN-I-amplified antibody (32, 66–68). The renal tissues of lupus-prone mice clearly showed IFN-I signatures, and IFN-I could trigger autoantibody production, such as anti-ds DNA Ab production, in pristane-induced lupus mice and NZB/W F1 lupus mice (69, 70). The importance of IFN-I downstream molecules in the immunopathogenesis of lupus nephritis has also been addressed by many researchers. In pristane-induced lupus mice and MRL/lpr mice, the deletion of NLRP12, a negative regulator of the immune response and IFN-I signaling, resulted in increased autoantibody production, glomerular IgG deposition, and monocyte recruitment in the kidney, leading to deteriorated renal function (71). The results also revealed that the protective effects of NLRP12 in lupus nephritis were mediated in an IFN-dependent manner (71). In addition to the upregulated expression of many molecules in immune cells and renal biopsy tissues, inhibitors that specifically or nonspecifically target the IFN-I signaling pathway, such as the enhancer of zeste homolog 2 (EZH2) inhibitor GSK126 or DZNep, exert therapeutic effects by prolonging survival, reducing anti-dsDNA Ab levels, and improving renal conditions in NZB/W F1 mice (72). In addition, blockade of the transcription factor IFN regulatory factor 5 (IRF5) protects against lupus nephritis by reducing serum anti-dsDNA Ab titers, attenuating kidney pathology and improving survival in MRL/lpr mice and pristane-induced lupus mice (73). Furthermore, conditional Irf5 deletion and pharmacological blockade of IRF5 after disease onset effectively inhibited disease progression and suppressed renal dysfunction (74). The treatment with the STAT3 inhibitor Stattic in MRL/lpr mice delayed the onset of proteinuria and reduced the production of anti-dsDNA Abs and inflammatory cytokines (75). Moreover, Zagury et al. demonstrated that the vaccination with an IFN-α kinoid comprising a mixture of keyhole limpet hemocyanin and murine IFN-α with aldehydes and eliciting anti-IFN-α antibody response resulted in a significant reduction of proteinuria, immune complex deposition in kidneys and death triggered by a recombinant IFN-α5 expressing adenovirus in NZB/W mice (76). Most notably, genetic approaches revealed that the deletion of IFN receptors in NZB mice resulted in a reduction in the levels of anti-erythrocyte autoantibodies, anti-DNA Abs, and erythroblastosis and the development of hemolytic anemia, kidney disease, and mortality (77). These animal studies suggest the critical roles of IFN-I signaling in lupus nephritis and suppressing IFN-I or IFN-I downstream signaling molecules conferred therapeutic benefits in animal models of lupus nephritis.

### 3.3 IFN-I signatures in kidneys of lupus patients

Kidney damage resulting from glomerulonephritis, SLE-like syndrome and thrombotic microangiopathy was reported in four patients receiving different IFN-I formulations (78). Several different glomerular injury patterns, such as collapsing glomerulopathy, membranoproliferative glomerulonephritis, and thrombotic microangiopathy, are common in IFN-I–related conditions, such as Type 1 interferonopathies, SLE and virus infection (53). Earlier studies by several researchers revealed a close association between the peripheral blood IFN-I signature and kidney biopsy findings in patients with lupus nephritis (33, 79). Patients who inadequately respond to therapy exhibited both a high IFN response signature and a high fibrotic signature in tubular cells of the kidney (80). Single-cell RNA sequencing analysis revealed a strong correlation between the IFN scores of tubular epithelial cells and several renal pathologies, such as cellular proliferation, tissue fibrosis, and poor therapeutic responses, in patients with lupus nephritis (81). Interestingly, in this report, the authors did not detect an association between IFN scores in immune cells, including B and T lymphocytes, natural killer cells and monocytes, and the clinical response (81). Wang et al. reported that the expression levels of an IFN-I downstream signaling molecule, IFN-inducible protein 16 (IFI16), which colocalizes with renal cells in the glomerulus and tubulointerstitium of the kidney in lupus nephritis patients, were correlated with pathological indices, disease activity and clinical prognosis (82). Importantly, IFI16 expression is significantly greater in kidneys of patients with lupus nephritis than in those of patients with various kidney diseases, such as minimal change disease, IgA nephropathy, and diabetic kidney disease (82). Furthermore, in kidney biopsy tissues from patients with SLE, differential expression of IFN-α2 and IFN-β transcripts was observed in patients with proliferative forms of lupus nephritis (class III/IV) compared with patients with membranous nephritis and control kidneys, suggesting differential effects of IFN-I subtypes in modulating the immunopathogenesis of different patterns of lupus nephritis (83). Overall, strong IFN-I signals in kidneys of patients with lupus nephritis can generally and consistently be observed in both animal and human systems.

## 4 Therapeutics targeting IFN-I and IFN-I downstream signaling molecules in SLE

The involvement of IFN-I and IFN-I downstream molecules in the immunopathogenesis of various clinical manifestations and the potential therapeutic benefits of anti-IFN-I agents in animal studies led to investigations of their anti-IFN-I effects in patients with SLE (84, 85). In a Phase IIb trial enrolling 431 moderate to severe active SLE patients with inadequate responses to standard-of-care treatments, sifalimumab, a fully humanized anti-IFN-α IgG_1κ_ monoclonal antibody, was effective in meeting the primary endpoint in determining the percentage of patients achieving an SLE responder index (SRI) response at week 52 (86). In addition to suppressing IFN-I-inducible gene expression, IFN-α inhibition with sifalimumab suppressed the mRNA expression of B-cell activating factor and the signaling pathways of tumor necrosis factor alpha (TNF-α), IL-10, IL-1β, and granulocyte−macrophage colony−stimulating factor (GM−CSF) in both the periphery and skin lesions (17, 87). Given the encouraging results from early-phase trials, Phase III studies are anticipated in the near future.

In a trial enrolling 231 SLE patients, treatment with the humanized anti-IFN-α IgG1 monoclonal Ab rontalizumab failed to meet the primary endpoint for reducing disease activity measured with the British Isles Lupus Disease Activity Group (BILAG)-2004 (primary endpoint) and SRI (secondary endpoint) at week 24 in all patients and in a subgroup of patients with high expression scores of IFN-regulated genes (interferon signature metric, ISM). Unexpectedly, in an exploratory analysis, rontalizumab treatment was associated with improvements in disease activity, reduced flares and decreased steroid use in SLE patients with low ISM scores (88).

Anifrolumab, an anti-IFNR1 mAb, appears to be the most successful anti-IFN-I regimen for treating patients with SLE. A Phase II trial enrolling 305 moderate-to-severe SLE patients treated with anifrolumab reported a significant improvement in the percentage of patients who achieved an SRI response at week 24, with a sustained reduction in the number of patients receiving oral corticosteroids in the low-dose (300 mg, *P* = 0.014) group but not in the high-dose (1000 mg, *P* = 0.063) group (89). A greater effective size in patients with a high IFN signature at baseline was also observed (89). A Phase III study (TULIP-1) of anifrolumab in 457 moderate-to-severe SLE patients failed to meet the primary endpoint measuring the proportion of patients achieving an SLE Responder Index (SRI-4) response at week 52, although the effects on reducing the corticosteroid dose, cutaneous lupus erythematosus disease area and severity index (CLASI) response, and BICLA response included as the secondary endpoints were achieved (90). Following the unsuccessful trial, the results from another Phase III trial (TULIP-2) enrolling 362 SLE patients that received monthly anifrolumab (300 mg) or placebo for 48 weeks demonstrated a significantly improved response rate at week 52 compared with placebo treatment, as measured using the BILAG-based Composite Lupus Assessment (BICLA) (91). Interestingly, TULIP-2 also revealed no striking difference in response in patients with a high IFN gene signature (48.0%, and 30.7% in the placebo group) and patients with a low interferon gene signature (46.7%, and 35.5% in the placebo group), although the percentages in both placebo groups were different (91). *Post hoc* analyses of pooled data from two Phase III trials (TULIP-1/TULIP-2) including 726 SLE patients confirmed the effectiveness of anifrolumab (300 mg monthly), especially for patients with high IFN gene signatures (92). Genome-wide RNA sequencing of whole-blood and protein data from SLE patients enrolled in these trials with anifrolumab (TULIP-1 and TULIP-2) suggested that anifrolumab could modulate multiple IFN-I downstream signaling pathways involving apoptosis and innate and adaptive immunity that are highly associated with SLE immunopathogenesis (93). The success of anifrolumab in reducing disease activity and corticosteroid usage led to its approval by the FDA in July 2021 for the treatment of SLE patients with moderate to severe disease (68, 91, 94).

The effectiveness of blocking the IFN-I downstream signaling molecules JAK in the treatment of SLE was examined. A total of 1655 SLE patients receiving the JAK1/2 inhibitor baricitinib at 4 mg or 2 mg for up to 3.5 years did not experience increased adverse events compared with those receiving placebo, yet the incidence of venous thromboembolism did not increase (95). A study enrolling 775 SLE patients with active lupus receiving either a 4 mg or 2 mg daily dosage of baricitinib in a Phase III trial failed to demonstrate its effectiveness in attaining the primary endpoint of measuring the proportion of SRI-4 responders at week 52 compared with those receiving placebo treatment (96). In contrast, in another Phase III study enrolling 760 patients with active SLE, daily baricitinib at a dose of 4 mg but not 2 mg achieved the primary endpoint compared with placebo treatment (97). However, baricitinib treatment did not affect glucocorticoid tapering or the time to first severe flare (97). A Phase II study enrolling 341 patients with moderate-to-severe active SLE revealed that the ABBV-599 high dose (elubrutinib, a selective Bruton tyrosine kinase inhibitor, 60 mg + the JAK1 inhibitor upadacitinib, 30 mg) or upadacitinib (30 mg) attained the primary endpoint, defined as the proportion of patients who achieved an SRI-4 score and a steroid dose ≤ 10 mg daily compared with placebo at week 24; however, the low dose of ABBV-599 (elubrutinib, 60 mg + Upadacitinib, 15 mg) and 60 mg of elsubrutinib alone failed to show effectiveness compared with placebo treatment (98)[(NCT03978520)]. In a Phase II study enrolling 363 patients with active SLE, 3 mg or 6 mg of the TYK2 inhibitor deucravacitinib twice daily achieved the primary endpoint of an SRI‐4 response at week 32 compared with placebo; however, 12 mg of deucravacitinib once daily failed to have a significant effect compared with placebo treatment (*P* = 0.08) (99). All clinical trials reported above examining anti-IFN agents and JAK inhibitors excluded patients with active, severe lupus nephritis or active neuropsychiatric SLE at enrollment.

## 5 Anti-IFN agents in lupus nephritis and lupus skin lesions

Several potential small-molecule inhibitors or biological agents that target the treatment of lupus nephritis have been discussed, including biologics such as belimumab, obinutuzumab, and anifrolumab; nonimmune-mediated renoprotective agents such as dapagliflozin and empagliflozin; and dual immunosuppressive and antiproteinuric compounds such as voclosporin (100). Regarding anti-IFN-I treatment, a Phase II double-blinded study that randomized 147 patients with Class III/IV lupus nephritis and examined the efficacy of anifrolumab (300 mg monthly) failed to meet primary endpoint changes in the baseline 24-hour urine protein−creatinine ratio at week 52, although numerical improvements in other endpoints, such as complete renal response, were noted (101). However, this trial also revealed that dosing with intensified regimens (900 mg intravenous anifrolumab for the first 3 doses before monthly 300 mg) was significantly better than merely basic regimens (300 mg monthly) or placebo treatment in achieving complete renal response and sustained glucocorticoid reduction (101). Encouragingly, the extension study, which lasted up to 104 weeks, confirmed the greater benefit for intensified regimens of anifrolumab in terms of complete renal response and corticosteroid tapering compared with basic regimens of anifrolumab or placebo. Moreover, the extension study also revealed a substantial improvement in the estimated glomerular filtration rate in both anifrolumab-treated groups (intensified and basic regimens) compared with the placebo-treated group (102).

A 2-year, randomized, double-blind, placebo-controlled, Phase III study is expected to enroll 360 participants to evaluate the efficacy and safety of anifrolumab as an added-to-standard therapy (consisting of mycophenolate mofetil and glucocorticoids) in patients diagnosed with active Class III or IV lupus nephritis (with or without concomitant Class V lupus nephritis), and the estimated study completion date is July 2028 (NCT05138133). In addition to this clinical trial (NCT05138133) and another trial NCT06015230 (for GR1603, an anti-IFNAR1 Ab) with vague description of exclusion criteria, the currently registered clinical trials, including trials NCT05620407 and NCT05617677 (for defravacitinib), NCT05440422 (for anifrolumab), NCT05843643 (for upadacitinib), NCT05856448 (for GLPG3667), NCT05879718 (for PF-06823859, an anti-IFN-β Ab), NCT06238531 (for gusacitinib, a JAK and SYK inhibitor), and NCT05966480 (for ESK-001, a TYK-2 inhibitor), exclude the participation of SLE patients with lupus neuropsychiatric manifestations or with active, severe Class III, and IV lupus nephritis that requires or may require treatment with cytotoxic agents or high-dose corticosteroids (**Table 1**).

**Table 1:** Currently ongoing trials examining biologics and small molecule inhibitors that target IFN-I or IFN-I downstream molecules in SLE.

Drug	Target	Design	Subjects No.	Measurement of primary endpoint	Completion (Estimated)	Clinicaltrials.gov ID
GR1603	IFNAR1	Phase Ib/II	120	Safety & SRI-4 response	2028-10-04	NCT06015230
Anifrolumab	IFNAR1	Phase III	346	Complete renal response	2028-07-07	NCT05138133
Anifrolumab	IFNAR1	Phase II	45	Cardio-ankle vascular index & pulse wave velocity & vascular inflammation in arteries	2024-08-01	NCT05440422
Deucravacitinib	Tyk2	Phase III	490	SRI-4 response	2027-12-17	NCT05620407
Deucravacitinib	Tyk2	Phase III	490	SRI-4 response	2027-12-17	NCT05617677
Upadacitinib	JAK1	Phase III	1000	BICLA response	2027-10-31	NCT05843643
Gusacitinib	JAK and SYK	Phase I	60	Adverse Events	2028-10-01	NCT06238531
PF-06823859	IFN-β	Phase II	48	IFN-I gene score (in CLE and skin lesions)	2026-11-18	NCT05879718
ESK-001	Tyk2	Phase II	388	BICLA response	2025-12-01	NCT05966480
GLPG3667	Tyk2	Phase II	180	SRI-4 response	2026-04	NCT05856448

IFNAR1, IFN-α receptor 1; JAK, Janus kinase; Tyk2, tyrosine kinase 2; SYK, spleen tyrosine kinase; SRI-4, SLE Responder Index-4; CLE, cutaneous lupus erythematosus.

BILAG, British Isles Lupus Disease Activity Group; BICLA, BILAG-based Composite Lupus Assessment.

The collective organ-wide analysis of TULIP trials revealed that the therapeutic effects of anifrolumab appeared to be more prominent in mucocutaneous lesions and other organ systems of SLE patients, such as the musculoskeletal system and the immunological system (92, 103). These effects were especially pronounced in patients with discoid lupus erythematosus and rituximab-resistant CLE (92). Anifrolumab efficacy was also found to be significant in reducing swollen joint counts but insignificant in reducing tender joint counts (103). A blood transcriptome analysis suggested that the suppression of a subset of ISGs may be adequate to achieve a therapeutic effect on skin lesions (104). Although only a limited number of patients (n=4) were analyzed, the effects of anifrolumab on mucocutaneous lesions in patients with SLE were also demonstrated in refractory CLE subtypes and lupus nonspecific mucocutaneous manifestations (105). Targeting and inhibiting IFN signaling pathways with anifrolumab was also very successful in SLE patients with difficult-to-treat skin lesions (106). Notably, in SLE patients with a high IFN-I gene signature and moderate-to-severe skin disease, the subcutaneous and biweekly administration of 150 mg or 300 mg of anifrolumab resulted in the neutralization of the IFN-I gene signature by 88.0% and 90.7%, respectively, suggesting that blocking IFN-I with anifrolumab is sufficient for treatment (107).

## 6 Findings challenging the therapeutic benefits of anti-IFN-I in lupus nephritis

Although most studies suggest the potential of IFN-I as a therapeutic target for lupus nephritis, observations from several studies raise concerns. The analysis of serological samples from 65 SLE patients revealed a positive correlation between IFN-I levels and the Systemic Lupus Erythematosus Disease Activity Index (SLEDAI) score and anti-dsDNA levels and inversely correlated with complement 3 (C3) levels. Notably, the significant correlation was mainly in nonrenal systems, such as skin lesions (p=0.0002), and the correlation between IFN-I levels and lupus nephritis was insignificant (p=0.0669) (108). A retrospective analysis of 40 treatment-naïve SLE patients revealed no significant differences in serum IFN activity between patients with and without the renal domain and among the different subtypes of lupus nephritis, although a correlation existed between serum IFN activity and fever, hematologic disorders, and mucocutaneous manifestations (109). Furthermore, the examination of tubular cells from patients with lupus nephritis via single-cell RNA sequencing analysis revealed no significant correlations between IFN response scores and several clinical parameters, such as the urine protein-to-creatinine ratio at the time of biopsy, chronicity index scores (p = 0.83), and activity index scores (80).

Significantly increased expression of cytokines in the glomerulus, including IFN-γ, IL-12, IL-18 and IL-10, was correlated with disease activity indices in patients with lupus nephritis (110). In patients with SLE, the expression levels of genes that suggest a Th1 signal in the glomerulus, such as T-bet, IFN-γ and IL-2 genes, correlate with lupus activity markers, such as serum complement levels and anti-dsDNA Ab titers (110). The significance of T cells in lupus nephritis was further confirmed by observing that the characteristics of SLE in NZB/NZW mice induced by IFN-α, such as anti-DNA Ab production and the development of nephritis, depend on the presence of CD4+ T cells (111). Masutani et al. used immunohistochemical approaches to examine renal tissues of patients with lupus nephritis Class V and IV and minor glomerular lesions. They primarily found increased infiltration of CD68+ macrophages, CD3+ T cells, and IFN-γ-positive cells and a small number of IL-4-expressing T cells, which supports the predominance of the Th1 immune response in the lesion (112). Fava et al. analyzed the patterns of 1000 urine protein biomarkers in 30 patients with active lupus nephritis and reported that IFN-γ, but not IFN-I, which is mostly produced by infiltrating CD8^+^ and NK cells, is the main IFN in lupus nephritis (113). A network meta-analysis of the effectiveness of biological agents, including rituximab, abatacept, belimumab, anifrolumab, obinutuzumab, ocrelizumab, and low-dose IL-2, in 1,480 patients with lupus nephritis revealed that a significant proportion of patients achieving complete remission had received low-dose IL-2, obinutuzumab, rituximab, and belimumab (114). Among these, the low-dose IL-2 treatment arm had the highest percentage of patients attaining complete remission and a lower risk of serious adverse events (114). Altogether, these studies highlight the important roles of T cells stimulated by factors other than IFN-I in the pathogenesis of lupus nephritis.

Increased circulating neutrophil transcripts and the results of immunohistochemistry analysis of kidney samples from patients with lupus nephritis suggest the involvement of neutrophils in the immunopathogenesis of lupus nephritis (115). Similarly, granulocyte infiltration was also detected in kidneys of MRL/lpr mice, and treatment with the granulocyte inhibitor avacopan inhibited granulocyte infiltration and reversed renal conditions (14). Nevertheless, given that NETosis-mediated delivery of nucleic acid immunogens contributes to the production of IFN-α and damage to the glomerulus and renal tubules (83, 116, 117), the genetic depletion of peptidyl arginine deiminase type IV (PAD4), which eliminates NETosis, has no effect on reducing anti-DNA Ab titers or preventing kidney damage in MRL/lpr mice (118, 119).

Brohawn et al. applied whole-transcriptome array analyses to examine the IFN-I gene signature (IFNGS) of baseline blood samples from 681 moderate-to-severe SLE patients reported in two Phase IIb trials (38). The patients were simply allocated to either the IFNGS-high or the IFNGS-low population. The authors reported that whole-blood samples from SLE patients with high IFNGS expression were enriched with several inflammation-associated signaling markers, such as CD40L, CXC cytokines, TLR8-mediated monocyte activation, major histocompatibility complex class I, and the plasma cell gene expression signature, compared with patients with low IFNGS expression (38). Interestingly, when single-cell RNA sequencing was used to examine kidney tissues from patients with lupus nephritis, Arazi et al. reported that a high IFN response score was detected mainly in some populations of B cells and CD4^+^ T cells but not in kidney epithelial cells; however, B cells and CD4^+^ T cells also presented lower IFN response scores, suggesting spatialized localization of the IFN-I signal (30). Subsequent studies from these authors demonstrated that the IFN response is mainly an extrarenal process (30). These studies suggest that many potential factors or cellular populations, such as CXC cytokines, neutrophils and Th1 cells, may be also important in causing lupus nephritis as indicated above. Furthermore, the molecular heterogeneity of lupus nephritis may significantly affect patient outcomes in clinical trials using anti-IFN-I therapy.

In addition to IFN-α, the examination of blood and kidney samples from SLE patients revealed a positive correlation between IFN-β(+) B cells and anti-Sm, anti-DNA, and immune complex deposition in the glomerular basement membrane; active glomerular lesions with fibrocellular crescents; chronic glomerular lesions with segmental sclerosis; and a membranous pattern of renal damage (120). Nevertheless, IFN-β gene deletion does not affect disease progression, including autoantibody production, hemolytic anemia, kidney disease and mortality, in NZB mice (121). Unexpectedly, the administration of IFN-β to MRL-lpr mice with mild and advanced disease has shown beneficial effects by prolonging survival, reducing disease activity in clinical and histological analysis, and attenuating the production of serological parameters, such as autoantibodies and cytokines (122). Because anifrolumab treatment attenuates both IFN-α and IFN-β signaling, its therapeutic effects on lupus nephritis may be antagonized by the protective effects of IFN-β in lupus nephritis. Mejia-Vilet et al. analyzed more than 110 genes in kidney tissues at the time of diagnosis and at the time of flare-up in patients with lupus nephritis. These authors reported heterogeneity in immune-related gene expression, and the expression of half of the genes clustered when comparing the first biopsy with the repeated biopsy of kidney tissues. Interestingly, the expression of eight IFN-α-controlled genes was significantly greater in the diagnostic kidney tissues than in the flare biopsy samples in all patients. In contrast, the expression of nine TNF-α-controlled genes appeared to be greater in flared kidney tissues than in diagnostic kidney tissues (123). Studies from Petri et al. examining longitudinal changes in ISG expression in peripheral blood revealed an overall association between increased IFN response scores and increased disease activity in SLE patients; however, no significant changes in IFN response scores were detected between paired baseline samples and disease flare samples (124).

Studies have also suggested that the production of IFN-α may not be detrimental to some organ systems. For example, the production of IFN-α in the gut following commensal bacterial infection can be protective by inducing IL-27 in DCs and driving IL-27Rα signaling to ensure Foxp3+ Tregs and maintain immune tolerance (125). Finally, many critical immunopathogenic mechanisms demonstrated in both *in vitro* and *in vivo* studies may not be adequately blocked by anti-IFN-I treatment (30, 68, 126). Taken together, these factors can significantly hamper the therapeutic benefits of anti-IFN-I agents in patients with lupus nephritis.

## 7 Perspective

In the first part of this review, we addressed the important roles of IFN-I in the immunopathogenesis of SLE from various aspects suggesting the strength of developing anti-IFN-I to achieve the therapeutic benefits that may potentially outweigh the currently available treatments for patients with SLE. Unexpectedly, the anti-IFN-I treatment with anifrolumab was not working as “originally anticipated” in patients with lupus nephritis although its effectiveness in various manifestations, especially the difficult-to-treat skin lesions, is promising. We then must carefully look back and try to find some details that are likely to be missed along the way of developing anti-IFN-I treatment for SLE patients and pay more attention to re-evaluate immunopathogenic mechanisms of lupus nephritis. Finally, if anti-IFN-I is not sufficient to achieve the goal as we originally expected, by adding additional therapeutic regimens, we may still have chance to achieve what we expect to have for the best and optimized therapeutic strategies for patients with lupus nephritis.

Appropriate patient stratification and efficient disease severity monitoring via the application of high-throughput technologies may optimize personalized medicine and provide biologics that are more efficiently used to treat SLE patients with major organ involvement, especially those with lupus nephritis (127). Compared with csDMARDs, the administration of biologics such as anifrolumab is clearly accompanied by a higher cost, is more inconvenient to deliver, and is more difficult to produce given the complicated procedures of manufacturing products. Therefore, biologics are anticipated to have much better therapeutic effects on the very challenging conditions of SLE, such as lupus nephritis, and fewer adverse events than csDMARDs. Belimumab, which targets B-cell activation, has been introduced to the market; however, it is used as an add-on therapy under strong background treatment, and the effects of belimumab on a crucial part of the disease, lupus nephritis, are not very promising. In recent years, a new powerful synthetic DMARD, voclosporin, has been proven to be effective for treating lupus nephritis under the background treatment with rapidly tapered low-dose oral steroids and mycophenolate mofetil (MMF) (128, 129). Accordingly, if biologics are not superior to synthetic DMARDs such as MMF, voclosporin and tacrolimus in the treatment of lupus nephritis, the advancement of these biologics will be greatly limited in clinical practice.

Although pan-inhibition of IFN-I may have broader effects on immunomodulation than specifically inhibiting certain IFN-I downstream signaling molecules, these effects may not translate to additional clinical benefits for certain conditions, such as lupus nephritis. Notably, the activation of IRF5 and ISGs in the active and remission phases of SLE suggests that the therapeutic effects of inhibiting IRF5 are better than those of full inhibition of IFN-I signaling in Lyn-deficient lupus mice (74). This study suggests the potential of identifying IFN downstream molecules as therapeutic targets rather than complete inhibition of IFN-I to optimize therapeutic benefits in patients with lupus nephritis (74). More studies are needed to delineate the differential and potential roles of individual IFN-I downstream signaling molecules in lupus nephritis. Moreover, identifying the distinctive roles of IFN-I downstream signaling molecules in different classes of lupus nephritis will also be interesting. Importantly, given the heterogeneity nature of SLE, the most appropriate and optimized therapeutic strategies may need to include a combination of therapeutics targeting different molecules rather than one only.
